# Investigating the Role of Miscibility in Hydrogenated Dicyclopentadiene Resin/Polymer Blends: A Molecular Dynamics Study

**DOI:** 10.3390/polym18050594

**Published:** 2026-02-28

**Authors:** Anastassia N. Rissanou, Rohit Ghanta, Manolis Doxastakis, Vagelis Harmandaris

**Affiliations:** 1 Institute of Theoretical and Physical Chemistry, National Hellenic Research Foundation, 48 Vassileos Konstantinou Ave., 11635 Athens, Greece; 2 Department of Chemical and Biomolecular Engineering, University of Tennessee, Knoxville, TN 37996, USA; rghanta@vols.utk.edu (R.G.); edoxasta@utk.edu (M.D.); 3 Computation-Based Science and Technology Research Center, The Cyprus Institute, Nicosia 2121, Cyprus; harman@uoc.gr; 4 Department of Mathematics and Applied Mathematics, University of Crete, 70013 Heraklion, Greece; 5 Institute of Applied and Computational Mathematics, Foundation for Research and Technology Hellas, IACM/FORTH, 70013 Heraklion, Greece

**Keywords:** H-DCPD resin, SBR, PI, miscibility, molecular dynamics

## Abstract

The behavior and properties of polymer–resin blends are critical for the design of advanced polymeric systems in a wide range of applications. In this work, we present an atomistic molecular dynamics study of the effects of hydrogenated dicyclopentadiene (H-DCPD) resin on the structural, dynamical, and viscoelastic properties of polymer matrices. Two different systems are examined: linear 1,4-cis polyisoprene (PI) and a four-component styrene–butadiene rubber (SBR) copolymer. The results reveal lower miscibility of H-DCPD resin in PI compared to that in SBR, as evidenced by the formation of resin-rich domains, revealed by the magnitude of local peaks in resin–resin radial distribution functions. Temperature-dependent analysis shows that structural and dynamical properties are most sensitive at ambient conditions (T = 300 K, P = 1 atm), with PI exhibiting significantly reduced molecular mobility due to its proximity to the glass transition temperature. This restricted dynamics directly influences the viscoelastic response, leading to increased structural rigidity upon resin addition. Furthermore, the effect of resin concentration is systematically assessed, demonstrating that, while 17 vol% resin has a negligible impact on both systems, increasing the concentration to 34 vol% in PI, results in pronounced changes in its structural and viscoelastic behaviors. Overall, this study highlights atomistic molecular dynamics simulations as powerful predictive tools for the rational design of resin–elastomer blends.

## 1. Introduction

Thermoplastic elastomers (TPEs) are a class of materials designed to combine the elastic resilience of crosslinked rubbers (thermosets) with the processing flexibility of thermoplastics. Although both thermosets and thermoplastic polymers exhibit rubber-like behavior, they differ fundamentally in their molecular architecture and thermal response. Thermosets possess irreversible covalent crosslinks, which preclude remolding, whereas TPEs rely on reversible physical crosslinks to allow melt reprocessing and recyclability [1]. In both systems, the macromolecule crosslinking process plays a crucial role in defining the mechanical strength, the elasticity, and the durability of the material. Industrially, this network formation is applied to elastomers such as natural rubber (NR), polyisoprene (PI), and styrene–butadiene rubber (SBR), where engineered networks enable materials to recover from mechanical deformation and be resilient [2,3].

To design advanced elastomers, it is critical to understand how polymer networks form and how their structure governs performance. Atomistic simulations offer powerful and complementary tools for experiments, providing the necessary molecular-level insights into polymer behavior. Molecular Dynamics (MD) and Monte Carlo (MC) simulations have proven invaluable for exploring the effects of crosslink density, linkage types, and segmental mobility on the mechanical and dynamic properties of rubbers [4,5,6,7,8,9,10]. Studies on polymers such as polybutadiene (PB), PI, and SBR have demonstrated how subtle variations in chain architecture and intermolecular interactions can dramatically alter the morphology of the blend and the response to stress [11,12,13].

The role of resin molecules in elastomeric formulations is multiple. Certain resins actively participate in the crosslinking process, either by creating chemical bonds or by improving the dispersion of vulcanizing agents [14,15,16]. Low-molecular-weight resins, especially in adhesives, act as tackifiers, altering polymer flow and structure, based on their compatibility with the polymer matrix [17]. The degree of resin miscibility dictates whether homogenous blending or microphase separation occurs, which, in turn, has distinct effects on the material’s viscoelastic behavior and mechanical performance [18,19].

Investigation of the effects of different resins on the structure, dynamics, and curing characteristics of styrene–butadiene rubber compounds through various experimental techniques showed that resins influenced vulcanization kinetics and crosslink densit [14]. The addition of resins led to a decrease in crosslink density, suggesting that resins can interfere with crosslinking efficiency during vulcanization. This was found to be particularly pronounced with resins containing polar and acidic groups, which can deactivate curatives. Another experimental study investigates the impact of tackifying resin Kristalex™ 5140 on rubber compounds using solid-state NMR techniques [20]. The polymers involved are natural rubber, isoprene rubber, butadiene rubber, and styrene/butadiene rubber. The resin molecules mentioned include α-methylstyrene/styrene resin and various oligomeric hydrocarbon resins. It was found that resin addition increases the glass transition temperature (T_g_) and alters the polymer dynamics. Segmental dynamics slows down with the presence of resin, but collective polymer dynamics remains unaffected, while the resin is well-mixed with the rubber matrix, maintaining rigidity. Moreover, it was observed that the addition of resin has a more pronounced effect on cured samples because of the formation of crosslinks, which restrict further molecular mobility. In a recent study, all-atom MD simulations were utilized to investigate the glass transition temperature and compatibility of rubber/silica and rubber/resin composites [21]. The compatibility between rubber and different resins was found to affect T_g_ after blending by influencing the molecular interactions and mobility of the rubber chains. Good compatibility typically led to a higher T_g_ as a result of restricted movement of the rubber chains, whereas poor compatibility resulted in a lower T_g_, making the material softer and more plastic. The specific impact on T_g_ depended on the type, content, and molecular structure of the resin used. Moreover, the addition of silica to rubber increased T_g_ as a result of weak attractive interactions between silica and rubber molecular chains.

In elastomeric systems such as PI and SBR, the incorporation of hydrogenated dicyclopentadiene (H-DCPD) resin offers a promising route to tailor material properties. The rigidity and thermal stability of H-DCPDs significantly influence the morphology and stress distribution of the mixture [22]. Therefore, understanding its miscibility with various elastomers is essential to optimizing formulations for applications such as tires and flexible adhesives. In a recent work, Miccio et al. [23] utilized differential scanning calorimetry and broadband dielectric spectroscopy, and developed a predictive model for blend dynamics using the Gordon–Taylor equation and Adam–Gibbs approach, enabling accurate predictions of relaxation dynamics from minimal calorimetric data. Different polymer compounds, including polybutadiene rubber (PBD), styrene–butadiene rubber (SBR) and polyisoprene (PI), were studied, while the resin used in the mixtures was a hydrogenated DCPD/C9 resin with an aromatic level of 10%. Various concentrations of this resin were mixed with the polymers to analyze their interactions and dynamics. In the studied blends, the resin and the corresponding polymers were considered miscible. The presence of resin in the polymer blends led to slower segmental dynamics, as indicated by a shift in the alpha relaxation peak to lower frequencies with increasing resin content.

Although previous simulation studies have explored general resin/elastomer compatibility, the current work expands this scope by providing a comparative atomistic investigation of H-DCPD resin blended with PI and SBR. We focus on uncrosslinked systems to explore the direct effect of the resin on the properties of the polymer matrices. By analyzing molecular packing, dynamic behavior, and stress response, this study reveals how differences in miscibility shape the structural and viscoelastic characteristics of each system. The choices of PI and SBR are particularly strategic, as these polymers differ in backbone flexibility, polarity, and segmental dynamics, factors that critically influence miscibility with H-DCPD resin. Furthermore, they are the main constituents of different components of automobile elastomers; for example, polyisoprene (PI) is typically used in tire tread, whereas styrene–butadiene rubber (SBR) constitutes the main body. Through detailed analysis of radial distribution functions, mean square displacements, and shear relaxation moduli, this study reveals how variations in resin compatibility lead to different microstructural arrangements and viscoelastic responses. The effects of temperature and composition are also explored. These findings not only validate and extend previous simulation frameworks, but also offer predictive guidance for tailoring resin–elastomer formulations in applications such as adhesives, sealants, and tire compounds using atomistic simulation as a predictive tool.

## 2. Model and Systems

**Figure 1:**
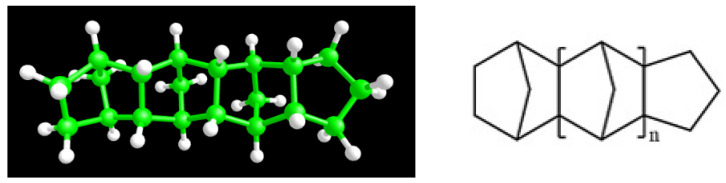
Model for hydrogenated dicyclopentadiene (H-DCPD) 3-mer resin molecular structure.

We study polymer–resin blends with two different polymer matrices (PI and SBR). SBR chains are copolymers comprising four components, the 1,4-cis, the 1,4-trans, and 1,2-vinyl isomers of polybutadiene (PB) and styrene (PS). In this study, the SBR polymer chains comprise 30 monomers, varying in both their sequence and the relative proportion of component units, resulting in an average composition in the number of monomers equal to cis:trans:vinyl:styrene = 26:33:26:15. A well-validated united atom model was used for the description of the SBR chains, where all bonded and non-bonded interactions for the PB units are described in our previous works [12,13,24]. For the styrene monomers, the force field was based on the TraPPE united atom model [25] with modifications and additions for the bonded, angular, and dihedral interaction types present in the SBR chain, reported in our previous publications [10,26]. For PI systems, an all-atom model for 1,4-cis PI was implemented, based on the force field that was extensively used and validated in an earlier study [27]. Each PI chain consisted of 30 monomers. A trimer of the H-DCPD resin molecule was modeled in an all-atom representation using the OPLS-AA force field parameters [28] obtained from the LigParGen server [29]. Interactions between polymer and resin molecules were parameterized through a spherically truncated 6–12 Lennard-Jones potential and standard geometric mixing rules with a cut-off distance of 1 nm for SBR systems and 1.4 nm for PI systems. The chemical formula, together with the model representation of the resin molecule, is presented in Figure 1. Systems with two resin concentrations (17 vol% and 34 vol%), similar to those used in elastomers, were prepared. For SBR blends, mixtures of 300 and 125 H-DCPD resin molecules respectively, with 96 SBR chains, were set up. In the PI blends, 232 and 464 H-DCPD resin molecules respectively, were mixed with 100 PI chains. Simulations were performed at T = 300 K (for SBR); 298 K (for PI) and T = 413 K to explore the effect of temperature. The starting configuration for SBR at 413 K was obtained from an equilibrated bulk system developed in our previous study [10]. To prepare the 300 K initial structure, the system was quenched from 413 K to 290 K at a cooling rate of 1.5 K/ns, followed by a 20 ns equilibration run at 300 K. Likewise, for PI, the starting configurations both at 413 K and 298 K were obtained from bulk systems simulated in a previous study by quenching from a high to low temperature at a cooling rate of 0.5 K/ns [27]. For both polymers, the corresponding bulk systems were simulated at the same conditions. The term “bulk” is used for pure polymer systems.

All systems were subjected to MD simulations using Gromacs software (GROMACS v. 2019.6) [30]. Long-range dispersion corrections were applied for both energy and pressure. Timesteps of 1.5 fs and 1 fs were employed for PI and SBR systems respectively, to ensure that simulations were efficient and stable throughout the long trajectories recorded. Relatively short NVT simulations (10 ns) were performed for all the resin–polymer systems for equilibration. Production NPT simulation runs were performed with the Berendsen barostat [31], maintaining pressure at P = 1 bar. The temperature was adjusted using the velocity-rescale thermostat [32] with a coupling constant of 0.5 ps.

## 3. Results and Discussion

The comparison between the SBR and PI mixtures with H-DCPD resins is made at two different temperature values (300 K and 413 K), at a fixed resin concentration, w_resin_ = 34 vol%, and at two different concentration values (w_resin_ = 17 vol% and w_resin_ = 34 vol%), at a fixed temperature, 413 K. Results of the corresponding bulk systems are provided for comparison in all cases. Notably, this comparison involves a 1,4-cis PI linear homopolymer and a -component SBR copolymer, which represent two inherently different polymer matrices.

### 3.1. Structure and Miscibility of Polymer–Resin Blends

At 34 vol% resin concentration, SBR–H-DCPD blends are denser than PI–H-DCPD blends at both temperature values, as shown in Table 1, following the trend of the corresponding polymer melts.

In all systems, adding resin molecules decreases the density of the mixtures compared to their respective bulk systems. The decrease is approximately 5% at room temperature, becoming slightly larger at 413 K (about 6% for SBR and 7% for PI). The small differences observed in density suggest corresponding differences in polymer dynamics and, potentially, in their resulting mechanical responses. It is worth noting that the room-temperature values used for the two polymers were marginally offset (298 K for PI and 300 K for SBR), but this difference is considered to have a negligible impact.

**Figure 2:**
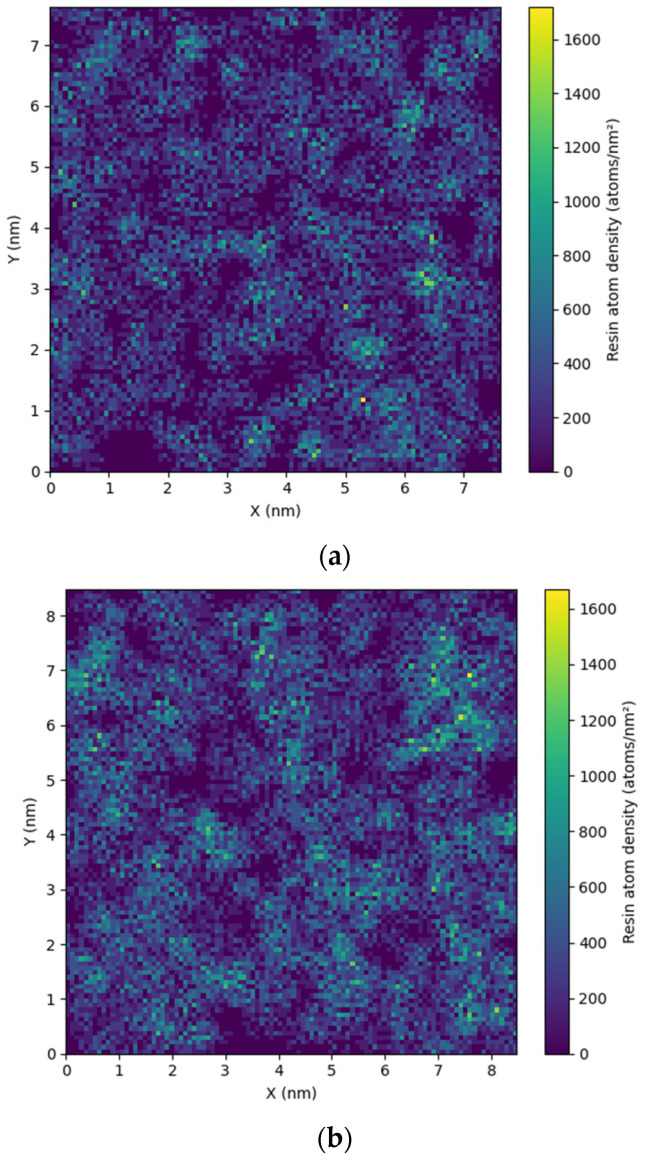
Number density of the resin atoms (#atoms/nm^3^) at 413 K in (**a**) for the SBR—H-DCPD mixture and (**b**) for the PI—H-DCPD mixture.

Another important factor that affects the mobility and elastic properties of the system is the miscibility of the two blended components. To investigate this, we initially performed a detailed analysis of the spatial distribution in the mixture by computing a “density tomography”, using 2D cross sections (slices) of the simulation domain. The side of the cubic box was equal to 7.5 nm for SBR and 8.5 nm for PI, and 100 bins were used in the z-direction, with each slice being 0.07 nm for the SBR and 0.08 nm for the PI system. In Figure 2, the density of the resin atoms in the mixture at 413 K is depicted; different values of the density are shown with different color coding. Although the overall picture suggests relatively good mixing, small regions enriched with H-DCPD are evident, particularly in the PI system, indicating reduced miscibility in this blend compared to the SBR mixture. As temperature decreases and the systems approach their glass transition (T_g_), phenomena such as reduced miscibility or kinetic arrest issues become increasingly dominant. This trend is particularly notable in the PI system (Figure S2).

However, the potential presence of temperature-driven phase separation, such as Lower Critical Solution Temperature (LCST), may influence the mixing behavior of resin–rubber systems. Experimental studies on natural and synthetic rubber blends with tackifiers and terpene or rosin resins have reported both LCST- and UCST-type phase behaviors, depending on the specific chemical interactions and composition of the blend [33,34]. More recently, temperature gradient experiments have further highlighted the sensitivity of tackifier segregation to thermal conditions in SBR matrices [35]. To investigate this possibility, we conducted additional simulations at 600 K for both systems, and the resulting density profiles are provided in Figure S3. At this elevated temperature, the SBR system exhibits clear cluster formation, indicative of reduced miscibility, while the PI system shows a similar, albeit less pronounced, effect. Further quantification is provided by the intermolecular pair radial distribution function between polymer and resin atoms, shown in Figure S4 for both mixtures. A comparison of the curves at 413 K and 600 K reveals reduced affinity at the higher temperature, characterized by lower peak intensities and a shift toward longer distances in both systems. These results suggest that both mixtures likely possess an LCST above the initially investigated temperature range. Consequently, the subtle miscibility differences observed at 413 K can be attributed to the distinct polymer matrices, with mixing becoming increasingly hindered at lower temperatures.

**Figure 3:**
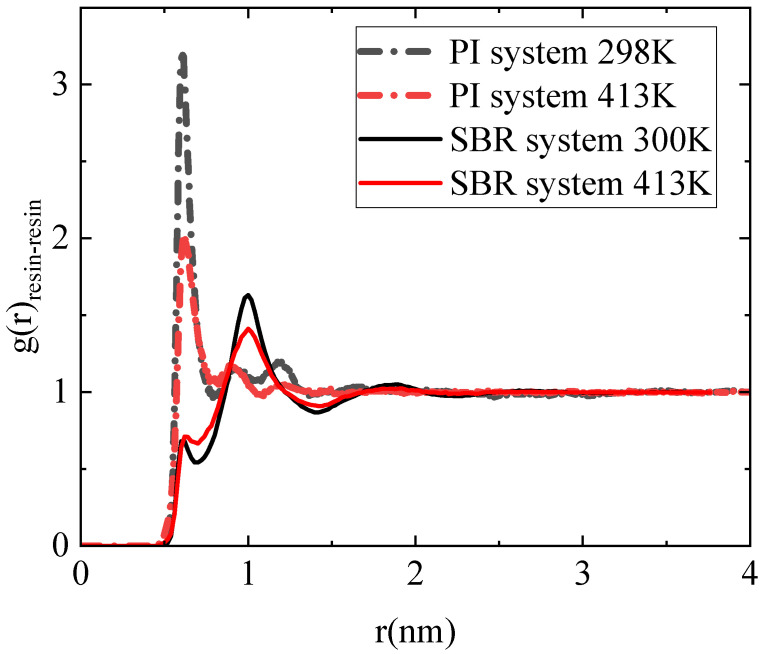
Pair radial distribution function calculated between the center mass of the resin molecules (g(r)_resin-resin_) for both PI–resin and SBR–resin mixtures at 300 K and 413 K.

A more detailed analysis of the structure and the miscibility of the polymer–resin blends can be provided by the pair radial distribution function (RDF), g(r), between the different components in the model polymer–resin blends. Data for the RDF between the centers of mass of the resin molecules, g(r)_resin-resin_, for both polymer–resin blends are shown in Figure 3. There is an obvious difference in the first peak of RDF (g(r)_resin-resin_), which is considerably higher and at shorter distances in the PI matrix. The first peak is located ~0.6 nm in the PI blend at both temperature values, although its intensity is attenuated at the higher temperature. In contrast, in the SBR mixture, g(r)_resin-resin_ exhibits its first main peak at ~1 nm, which is also shorter at higher temperatures. In particular, in the SBR mixture, g(r)_resin-resin_ displays a much smaller peak (a peak precursor) at the short distance of ∼0.6 nm, corresponding to the location of the first main peak in the PI blend. This picture signifies a stronger tendency for the H-DCPD molecules to aggregate in the PI mixture, which corresponds to the small resin-rich regions observed more pronounced in Figure 2b.

**Figure 4:**
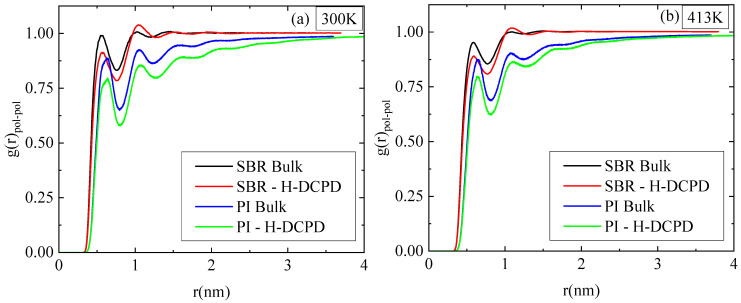
Total pair radial distribution function calculated between the center of mass of monomers (g(r)_pol-pol_) for PI and SBR polymer chains in the resin mixtures, together with the corresponding bulk curves at (**a**) 300 K and (**b**) 413 K.

Furthermore, the RDFs between the center of mass of the monomers, g(r)_pol-pol_, are presented in Figure 4 for the various polymer–resin mixtures and the two temperatures; as reference systems, the corresponding data for the bulk polymer systems are also shown. The obvious reduction in the height of the first peak in the g(r)_pol-pol_ ratio for the blends, relative to those of the bulk systems, suggests a slight decrease in the proximity between the polymer chains because of the presence of resin molecules. The findings are analogous for SBR and PI at both temperatures. When comparing the two polymers, PI consistently exhibits shorter peaks than SBR in all cases. This difference signifies that the PI chains are less densely packed than the SBR chains, both in the polymer mixtures and in their respective melts.

An interesting question concerns the potential effect of resin on the dimensions of polymer chains in the polymer–resin blends. To investigate this, we compute the average radius of gyration, R_g_, of polymer chains in the systems with and without resin; data are shown in Table 2. It is clear that the differences observed above, in the local environment (molecular packing) of polymer chains, do not alter the overall size of the polymer chains, as quantified by the R_g_. At 413 K, the polymer blend serves as a Θ-solvent for both SBR and PI. At the lower temperature of 300 K, SBR remains under Θ-solvent conditions, whereas PI exhibits a slight shift towards good solvent behavior. The effect of temperature points to a marginally increased chain dimension for PI under lower temperature conditions.

### 3.2. Dynamics and Viscoelasticity of Polymer–Resin Blends

**Figure 5:**
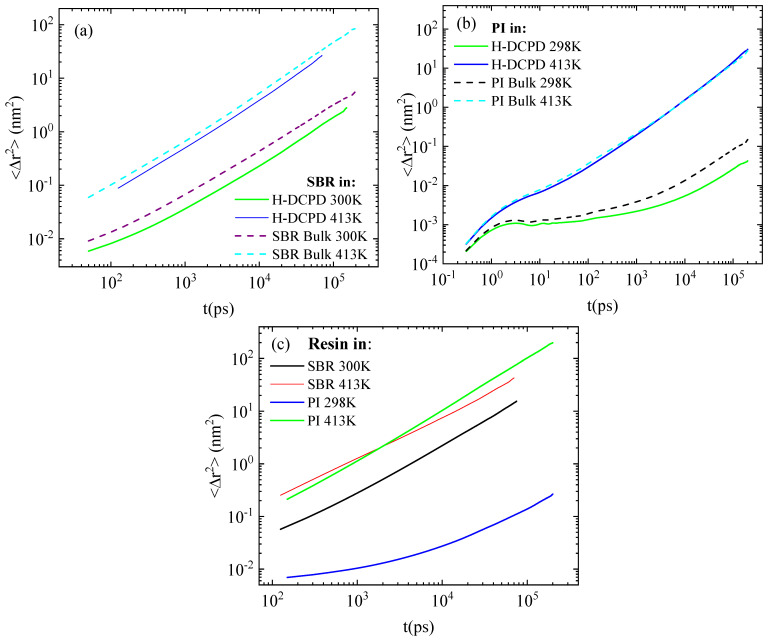
Mean square displacement of the center of mass of (**a**) SBR chains, (**b**) PI chains, and (**c**) H-DCPD resin molecules at both temperatures.

Moving on to dynamical properties, we explore the effect of H-DCPD resin molecules on the mobility of the polymer chains. The mean square displacement (MSD) of the center of mass of the polymer chains at low and high temperatures, along with the corresponding bulk curve, is presented in Figure 5a and Figure 5b, respectively. MSD is given by the formula $\langle {∆r}^{2}(t)\rangle =\frac{1}{N}\sum_{i=1}^{N}\langle {\left|r_{i}\left(t\right)-r_{i}\left(0\right)\right|}^{2}\rangle$, where *N* is the total number of chains in the system, and *r_i_*(*t*), *r_i_*(0) the positions of the center of mass of the ith chain at times *t* and 0 accordingly. Slower dynamics is observed in PI (Figure 5b) compared to SBR (Figure 5a) with larger differences, more than an order of magnitude, at lower temperatures. This is an important observation because it reveals that the glass transition temperature of SBR covers a wide range of values depending on its chemical composition and, most importantly, the weight percentages of styrene and vinyl. For styrene content < 15 wt%, the polymer is dominated by the cis/trans-1,4-butadiene units, which render the chain significantly more mobile than polyisoprene, which is hindered by the methyl group on its isoprene units [36]. The SBR system under study contains ~15 wt% styrene with a model T_g_ of approximately 260 K. The corresponding model T_g_ for the bulk system is ~245 K. Using the same cooling rate of 10 K/ns, we calculated the model T_g_ values for PI systems, which are ~300 K for the blend and ~265 K for the bulk system. The density as a function of temperature from the quenching runs is presented in Figure S1 for both the bulk systems and the blends. A small overestimation is observed in the calculated T_g_ for PI, compared to the experimental reported one for bulk system (~200 K)^24^, which can be attributed to the high cooling rate used [37,38]; this is a common issue in all atomistic MD simulations [39,40,41]. In simulations, the use of shorter chains and faster cooling rates creates two competing effects: shorter chains increase free volume and mobility, while rapid cooling, kinetically shifts the glass transition to higher temperatures [42]. This contrasts with experimental polymers, which typically consist of millions of monomers and exist well above their entanglement length. Specifically for PI, the short chain lengths lead to an increased chain-end concentration, which impacts both density and segmental dynamics [6,11], while dielectric relaxation spectroscopy suggests that local mobility approaches a chain-length-independent rate for molecular weights higher than 30,000 Da [43]. Based on the temperature difference between the test conditions and the respective glass transition temperatures, distinct variations in polymer dynamics are observed in bulk, and consequently in blends with, H-DCPD resin. SBR chains move considerably faster than PI at 300 K, while the difference is reduced at 413 K. The impact of the resin molecules is more pronounced at lower temperatures in both blends. At 413 K, the difference between the bulk and blended materials is negligible for PI, but slightly larger for SBR. The mobility of resin molecules is quantified in Figure 5c. The dynamics of H-DCPD molecules at 413 K is comparable across the PI and SBR mixtures. In contrast, cooling to 298 K/300 K induces a slowdown in dynamics, with this retardation being substantially greater in the PI mixture than in the SBR mixture, following the reduction in mobility of the PI chains.

**Figure 6:**
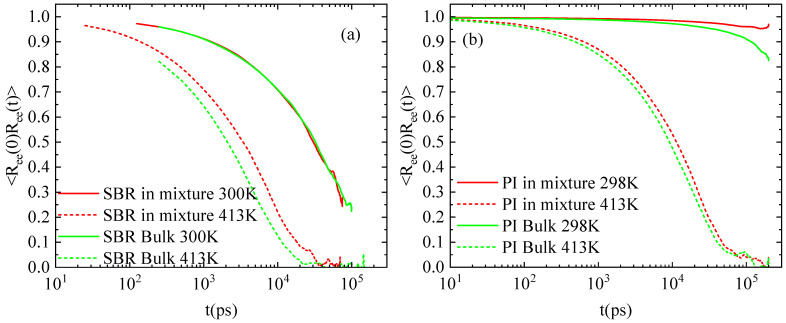
Autocorrelation function of P1 for the end-to-end vector of polymer chains in the mixture with the resin and the corresponding bulk systems at two different temperatures; (**a**) SBR, (**b**) PI.

Next, we investigate the rotational dynamics of polymer chains within the polymer–resin mixtures by probing the time autocorrelation function of the end-to-end vector of polymer chains, ${ACF}_{Ree}\left(t\right)=\langle R_{ee}(t+\tau )R_{ee}(t)\rangle /\langle R_{ee}^{2}\rangle$. Data for the time evolution of ACF_Ree_(t) are presented in Figure 6. The decorrelation of polymer chains is nearly unaffected by the presence of resins at low temperatures, but the resins cause a small retardation at the higher temperature. A large difference between SBR and PI is also evident at low temperature, rendering PI much slower, consistent with the corresponding chain dynamics. Almost no decorrelation is observed in the PI mixture at 298 K in 100 ns, whereas the corresponding bulk curve drops to ~0.8. The rotational dynamics of SBR at 413 K is also faster than that of PI, but by a smaller difference.

**Figure 7:**
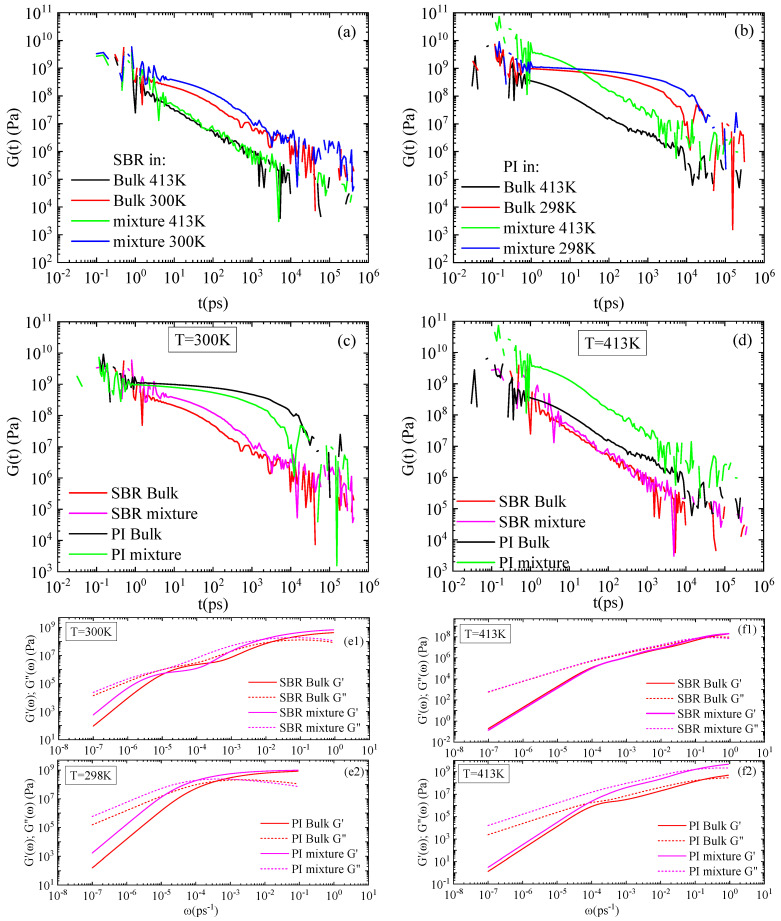
Shear relaxation modulus, G(t), for (**a**) SBR and (**b**) PI melts and mixtures with H-DCPD resin at 300 K and 413 K; comparison of the two polymer mixtures and the corresponding bulk systems at (**c**) 300 K and (**d**) 413 K; storage and loss moduli at 300 K for (**e1**) SBR and (**e2**) PI; storage and loss moduli at 413 K for (**f1**) SBR and (**f2**) PI.

The H-DCPD molecules affect the viscoelastic properties of the two blends in a different way. The viscoelastic behavior of the system is characterized by the shear-stress relaxation modulus:

$$G\left(t\right)=\frac{V}{3k_{B}T}\langle \sigma_{xy}(t)\sigma_{xy}(0)\rangle ,$$

where *V* is the volume of the system, *k_B_* the Boltzmann constant, and *σ_xy_* is the off-diagonal component of the stress tensor. Time-averaged correlations were computed over equilibrium trajectories of 200 ns. To improve computational efficiency and reduce memory requirements during long trajectories, we employed the multiple-tau correlator [44], which enables accurate calculation of time correlation functions on the fly with controlled error and minimal overhead [45]. This approach allows for accurate calculation of time correlation functions with controlled numerical errors. The temperature dependence of *G(t)* for SBR and PI is presented in Figure 7a and Figure 7b, respectively. The decrease in temperature leads to stiffer systems for both polymers. Adding resin to the SBR mixture slightly increases the overall stiffness of the system at 300 K, but has negligible impact at 413 K. However, the PI–resin blend exhibits a higher stiffness than that of the pure melt in both temperatures, with a more pronounced difference from the bulk at 413 K. Furthermore, all PI systems exhibit a higher degree of stiffness and structural resistance compared to SBR, surpassing the latter by approximately one order of magnitude, as observed in Figure 7c,d. This observation is consistent with the differences in temperature of the simulated systems from their corresponding glass transition temperatures, discussed for the dynamical properties, as well.

The storage and loss moduli, $\left(G’\left(\omega \right)=\omega \int_{0}^{\infty }G\left(t\right)\sin\left(\omega t\right)dt;\;G”(\omega )=\omega \int_{0}^{\infty }G\left(t\right)\cos\left(\omega t\right)dt\right)$, provide a more detailed picture of the viscoelastic behavior of the systems and are presented in Figure 7(e1,f1) for SBR and Figure 7(e2,f2) for PI. At short times, both SBR and PI display an almost balanced viscoelastic response, whereas the viscous behavior prevails for longer times at 413 K. The addition of resin molecules does not alter the aforementioned behavior in any of the blends, with negligible impact on SBR response. Similar correlations are observed at the low temperature, though dominance of viscosity is observed at longer timescales for SBR.

### 3.3. Effect of Resin Concentration on the Properties of Polymer–Resin Systems

**Figure 8:**
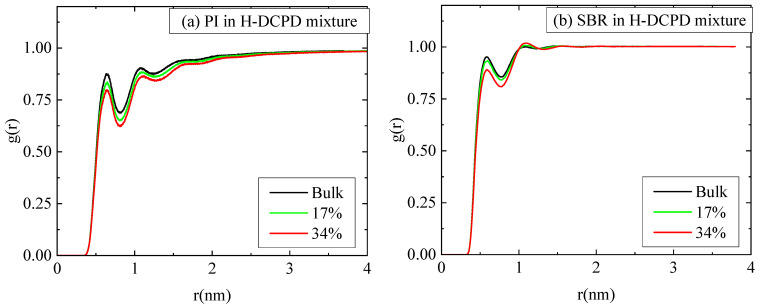
Pair radial distribution function between center-of-mass of monomers for (**a**) PI chains and (**b**) SBR chains in mixtures with resin (H-DCPD), together with the corresponding bulk curves at 413 K and two different resin concentrations.

In the next stage, we investigate the effects of resin (H-DCPD) concentration on the structural, dynamical, and viscoelastic properties of the model polymer/resin systems. Since content of resin of 34 vol% is universally classified as high [46], in the following, the effect of resin concentration on the blend is investigated comparing the above results of 34 vol% with a lower H-DCPD content of 17 vol% at T = 413 K in both polymer matrices. Figure 8 illustrates the influence of resin molecules on the spatial arrangement of polymer chains. This is quantified via the pair radial distribution functions, calculated between the centers of mass of the monomers for both polymers. A gradually weaker effect is observed as resin content is reduced from 34 vol% to 17 vol%, with the latter g(r) approaching the corresponding curves in melts.

**Figure 9:**
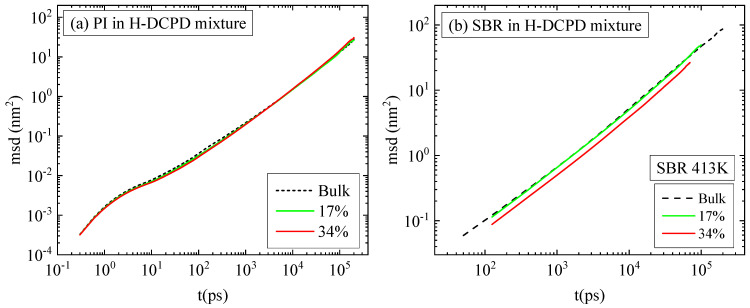
Mean square displacement for the center mass of polymer chains in resin mixtures with (**a**) PI and (**b**) SBR at 413 K and two different resin concentrations.

The mean square displacement of the center of mass of polymer chains, presented in Figure 9a and Figure 9b for the PI and SBR systems, respectively, quantifies the effect of resin concentration on the dynamics of polymeric chains. As expected, the lower the H-DCPD content, the weaker the effect. For PI polymer chains, the mixture with 17 vol% in resin has identical mobility as the PI melt, and a slight retardation is induced in the blend with 34 vol% in resin. Similarly, the low H-DCPD concentration has a negligible effect on SBR chain mobility; however, doubling the resin content results in a more pronounced retardation of dynamics compared to the PI systems.

**Figure 10:**
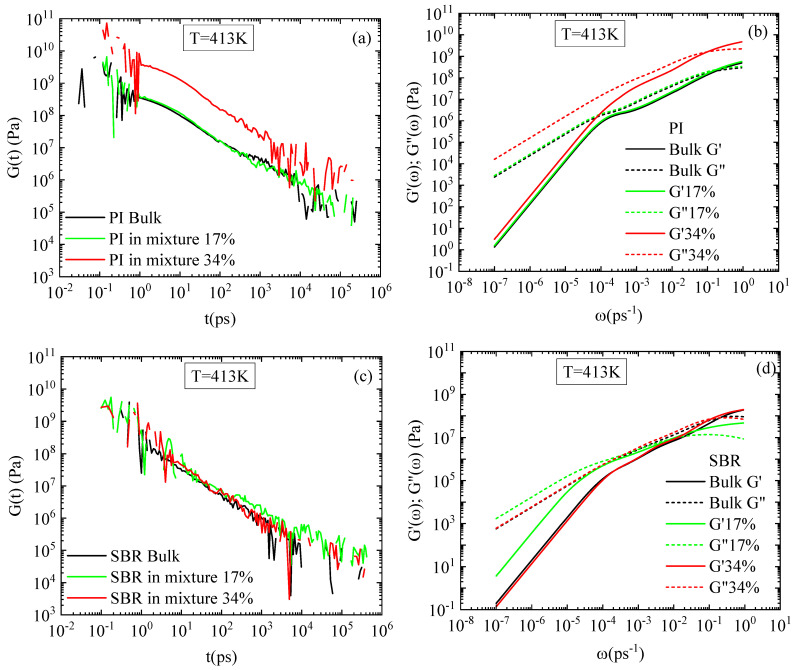
Shear relaxation modulus, G(t) for (**a**) PI, (**c**) SBR polymer melts and mixtures with H-DCPD; storage and loss moduli for (**b**) PI, (**d**) SBR polymer melts and mixtures with H-DCPD at 413 K and two different resin concentrations.

Finally, an analysis of viscoelastic properties was conducted for both concentrations of H-DCPD resin. The results reveal a significant concentration effect, highlighting a distinct divergence in behavior between the SBR and PI systems. The shear-stress relaxation modulus is presented in Figure 10a and Figure 10c for the PI and SBR mixtures, respectively, at both resin concentrations. At a loading of 17 vol% H-DCPD, the blends remain unaffected. However, increasing the resin concentration to 34 vol% has a negligible effect on G(t) for the SBR blend, whereas the PI blend exhibits a significant response, which indicates an increase in its stiffness. This behavior aligns with the difference observed between SBR and PI, especially in their respective blends at 34 vol%. Density remains largely homogeneous at low resin concentrations in both systems; however, increasing the H-DCPD content reduces miscibility within the PI blend (Figure 2), inducing rich-resin regions, which impact its viscoelastic properties. These observations are reflected in the corresponding storage and loss moduli. The SBR–H-DCPD system exhibits elastic dominance at short timescales, whereas it becomes more viscous at longer times (Figure 10d), and this behavior remains almost unchanged regardless of resin concentration. In the PI–H-DCPD system (Figure 10b), there is a significant difference from the corresponding bulk behavior at 34 vol% resin content. On the other hand, hardly any impact on the degree of stiffness and structural resistance is observed in the PI blend with the 17 vol% resin content, compared to the corresponding bulk system.

Low-frequency data suffers from insufficient statistics; a challenge also reflected in Figure 7. The use of the Green–Kubo formula (GK) for the calculation of the viscosity faces limitations at low frequencies. The reliance on the stress autocorrelation function, which decays very slowly, makes it very difficult to measure its long tail in detail, which is critical for characterizing low-frequency behavior.

## 4. Conclusions

The current work investigates the effect of H-DCPD resin in mixtures with two different polymer matrices, the 1,4-cis PI and the SBR, through molecular dynamics simulations. A linear 1,4-cis PI homopolymer and a four-component SBR copolymer were selected to represent two distinct polymer environments. These choices were specifically based on their contrasting physical properties, such as flexibility of backbone and segmental dynamics, which play a decisive role in how well they blend with the H-DCPD resin.

Two temperature values were used at a specific resin concentration to stress the effect of temperature on the properties of the mixtures. The reduced miscibility of PI with resin molecules compared to SBR, which is visualized through the atom density figures (Figure 2), is verified by the pair radial distribution function between the center mass of resin molecules within the blends (Figure 3). The higher peaks at shorter distances indicate a rich resin region in the PI blends. The influence of temperature on the structural properties is limited at 413 K, whereas more pronounced differences from the corresponding bulk systems are observed at room temperature. A similar trend is found in dynamics. However, a significant difference is observed in the mobility of the polymer chains in the two blends, followed by the mobility of the H-DCPD molecules. PI chains exhibit a markedly reduced mean square displacement compared to SBR chains, and the difference is particularly pronounced at 300 K. This is attributed to the values of the model T_g_, calculated for the two systems using the same, quite high, cooling rate of 10 K/ns (i.e., T_g_~265 K in the SBR blend; T_g_~300 K in the PI blend). The chains’ motion becomes more constrained as the simulation temperature approaches the polymer’s T_g_. The viscoelastic properties of blends are affected accordingly, where the presence of resin molecules induces stiffer behavior compared to the corresponding bulk systems. Although, in the SBR mixture, resin induces a higher shear relaxation modulus G(t) than that of its bulk counterpart at 300 K, the divergence between the mixture and the bulk in PI is more pronounced at 413 K. Temperature has no impact on the transition from solid to liquid behavior (G’ > G”). Both melts initially exhibit an elastic-dominant response that shifts toward a viscous-dominant regime at longer times.

Resin concentration has been examined using two different contents, 34 vol% and 17 vol%, of H-DCPD molecules in polymer blends at 413 K. Across this range of concentrations, the impact of resin on SBR blends is negligible for 17 vol%, and very small for 34 vol%, leaving the polymer’s intrinsic properties largely unaffected. Conversely, while 17 vol% resin has a similarly minor effect on PI, increasing the resin content to 34 vol% significantly alters the system’s structural and viscoelastic properties. The principal benefit of employing atomistic molecular dynamics in this study is its capacity to resolve fine-scale effects of the H-DCPD resin on both polymeric systems, which remain nearly imperceptible under experimental conditions. Overall, this study demonstrates the utility of atomistic simulation as a predictive methodology offering valuable insights into engineering specific resin–elastomer interfaces for high-performance applications.

## Figures and Tables

**Table 1:** Density of the model systems, *ρ* (gr/cm^3^).

T (K)	PI—H-DCPD	SBR—H-DCPD	PI Bulk	SBR Bulk
298/300	0.96 ± 0.10	1.01 ± 0.13	0.91 ± 0.07	0.97 ± 0.06
413	0.89 ± 0.09	0.94 ± 0.12	0.83 ± 0.02	0.89 ± 0.02

**Table 2:** Average radius of gyration of polymer chains, R_g_ (nm), in polymer–resin mixtures and in the corresponding bulk systems for two temperatures.

T (K)	PI—H-DCPD	SBR—H-DCPD	PI Bulk	SBR Bulk
298/300	1.45 ± 0.06	1.14 ± 0.08	1.33 ± 0.03	1.13 ± 0.03
413	1.36 ± 0.08	1.10 ± 0.10	1.34 ± 0.04	1.06 ± 0.05

## Data Availability

Dataset available on request from the authors.

## Supplementary Materials

The following supporting information can be downloaded at: https://www.mdpi.com/article/10.3390/polym18050594/s1, Figure S1: Density as a function of temperature from the quenching runs for (a) Bulk SBR, (b) Bulk PI, (c) SBR—Resin and (d) PI—Resin systems; Figure S2: Number density of the resin atoms (atoms/nm^3^) (a) at 300 K for the SBR—H-DCPD mixture and (b) at 298 K for the PI—H-DCPD mixture; Figure S3: Number density of the resin atoms (atoms/nm^3^) for (a) the SBR—H-DCPD mixture and (b) for the PI—H-DCPD mixture at 413 K the upper panel and at 600 K the lower panel; Figure S4: Intermolecular pair radial distribution functions between polymer and resin atoms for PI—H-DCPD and SBR—H-DCPD blends at 413 K and 600 K.

## References

[1] Spontak R.J., Patel N.P. (2000). Thermoplastic Elastomers: Fundamentals and Applications. Curr. Opin. Colloid Interface Sci..

[2] Kim D.Y., Park J.W., Lee D.Y., Seo K.H. (2020). Correlation between the Crosslink Characteristics and Mechanical Properties of Natural Rubber Compound via Accelerators and Reinforcement. Polymers.

[3] Marković G., Marinović-Cincović M., Samaržija-Jovanović S., Jovanović V., Budinski-Simendić J., Gutiérrez T.J. (2020). Crosslinking of Polymers: Rubber Vulcanization. Reactive and Functional Polymers Volume Two: Modification Reactions, Compatibility and Blends.

[4] Yan T., Wang K.-J., Zhao X.-Y., Gao Y.-Y. (2023). Effect of Cross-Linking Density on Non-Linear Viscoelasticity of Vulcanized SBR: A MD Simulation and Experimental Study. Int. J. Mol. Sci..

[5] Ghanta R., Papakonstantopoulos G.J., Domurath J., Polińska P., Burkhart C., Harmandaris V., Doxastakis M. (2025). Cross-Linked Polyisoprene Networks: Linker Architecture and Elastomer Properties. ACS Appl. Polym. Mater..

[6] Li W., Jana P.K., Behbahani A.F., Kritikos G., Schneider L., Polińska P., Burkhart C., Harmandaris V.A., Müller M., Doxastakis M. (2021). Dynamics of Long Entangled Polyisoprene Melts via Multiscale Modeling. Macromolecules.

[7] Grest G.S., Kremer K. (1990). Statistical Properties of Random Cross-Linked Rubbers. Macromolecules.

[8] Gula I.A., Karimi-Varzaneh H.A., Svaneborg C. (2020). Computational Study of Cross-Link and Entanglement Contributions to the Elastic Properties of Model PDMS Networks. Macromolecules.

[9] Guseva D.V., Rudyak V.Y., Komarov P.V., Bulgakov B.A., Babkin A.V., Chertovich A.V. (2018). Dynamic and Static Mechanical Properties of Crosslinked Polymer Matrices: Multiscale Simulations and Experiments. Polymers.

[10] Kallivokas S.V., Chazirakis A., Ghanta R., Rissanou A., Polińska P., Burkhart C., Doxastakis M., Harmandaris V. (2025). Elastic, Viscoelastic, Dynamic, Topological and Structural Properties of Crosslinked SBR through Atomistic Molecular Dynamics Simulations. Soft Matter.

[11] Doxastakis M., Mavrantzas V.G., Theodorou D.N. (2001). Atomistic Monte Carlo Simulation of Cis-1,4 Polyisoprene Melts. I. Single Temperature End-Bridging Monte Carlo Simulations. J. Chem. Phys..

[12] Behbahani A.F., Rissanou A., Kritikos G., Doxastakis M., Burkhart C., Polińska P., Harmandaris V.A. (2020). Conformations and Dynamics of Polymer Chains in Cis and Trans Polybutadiene/Silica Nanocomposites through Atomistic Simulations: From the Unentangled to the Entangled Regime. Macromolecules.

[13] Rissanou A., Chazirakis A., Polinska P., Burkhart C., Doxastakis M., Harmandaris V. (2022). Polybutadiene Copolymers via Atomistic and Systematic Coarse-Grained Simulations. Macromolecules.

[14] Pierigé M., Nardelli F., Calucci L., Cettolin M., Giannini L., Causa A., Martini F., Geppi M. (2024). Exploring the Effect of Resins of Different Origin on the Structure, Dynamics and Curing Characteristics of SBR Compounds. Polymers.

[15] Lee J.-Y., Park N., Lim S., Ahn B., Kim W., Moon H., Paik H., Kim W. (2017). Influence of the Silanes on the Crosslink Density and Crosslink Structure of Silica-Filled Solution Styrene Butadiene Rubber Compounds. Compos. Interfaces.

[16] Choi S.-S., Jang J.H. (1998). Tack Behaviours of P-t-Octylphenol Formaldehyde Resin with Rubber Using a Molecular Simulation. Polymer.

[17] Guo Y., Liu J., Lu Y., Dong D., Wang W., Zhang L. (2018). A Combined Molecular Dynamics Simulation and Experimental Method to Study the Compatibility between Elastomers and Resins. RSC Adv..

[18] Surendran A., Joy J., Parameswaranpillai J., Anas S., Thomas S. (2020). An Overview of Viscoelastic Phase Separation in Epoxy Based Blends. Soft Matter.

[19] Garate H., Morales N.J., Goyanes S., D’Accorso N.B., Parameswaranpillai J., Hameed N., Pionteck J., Woo E.M. (2016). Miscibility, Phase Separation, and Mechanism of Phase Separation of Epoxy/Block-Copolymer Blends. Handbook of Epoxy Blends.

[20] Pierigé M., Nerli F., Nardelli F., Calucci L., Cettolin M., Giannini L., Geppi M., Martini F. (2023). Influence of Resins on the Structure and Dynamics of SBR Compounds: A Solid-State NMR Study. Appl. Sci..

[21] Chen Q., Zhang Z., Huang W., Qu J., Zhang Q., Wu X., Zhang L., Liu J. (2024). Predicting the Glass Transition Temperature and Solubility Parameter between Rubber/Silica and Rubber/Resins via All-Atom Molecular Dynamics Simulation. Polym. Int..

[22] Lim D.-H., Do H.-S., Kim H.-J. (2006). PSA Performances and Viscoelastic Properties of SIS-Based PSA Blends with H-DCPD Tackifiers. J. Appl. Polym. Sci..

[23] Miccio L.A., Sill C., Wehlack C., Schwartz G.A. (2024). Connecting Dynamics and Thermodynamics in Polymer–Resin Cured Systems. Polymers.

[24] Smith G.D., Paul W. (1998). United Atom Force Field for Molecular Dynamics Simulations of 1,4-Polybutadiene Based on Quantum Chemistry Calculations on Model Molecules. J. Phys. Chem. A.

[25] Martin M.G., Siepmann J.I. (1998). Transferable Potentials for Phase Equilibria. 1. United-Atom Description of n-Alkanes. J. Phys. Chem. B.

[26] Harmandaris V.A., Adhikari N.P., van der Vegt N.F.A., Kremer K. (2006). Hierarchical Modeling of Polystyrene: From Atomistic to Coarse-Grained Simulations. Macromolecules.

[27] Ghanta R., Burkhart C., Polińska P., Harmandaris V., Doxastakis M. (2023). The Effect of Chemical Constitution on Polyisoprene Dynamics. J. Chem. Phys..

[28] Jorgensen W.L., Maxwell D.S., Tirado-Rives J. (1996). Development and Testing of the OPLS All-Atom Force Field on Conformational Energetics and Properties of Organic Liquids. J. Am. Chem. Soc..

[29] Dodda L., Cabeza de Vaca I., Tirado-Rives J., Jorgensen W. (2017). LigParGen Web Server: An Automatic OPLS-AA Parameter Generator for Organic Ligands. Nucleic Acids Res..

[30] Abraham M.J., Murtola T., Schulz R., Páll S., Smith J.C., Hess B., Lindahl E. (2015). GROMACS: High Performance Molecular Simulations through Multi-Level Parallelism from Laptops to Supercomputers. SoftwareX.

[31] Berendsen H., Postma J.P.M., van Gunsteren W., DiNola A., Haak J.R. (1984). Molecular-Dynamics with Coupling to An External Bath. J. Chem. Phys..

[32] Bussi G., Donadio D., Parrinello M. (2007). Canonical Sampling through Velocity Rescaling. J. Chem. Phys..

[33] Fujita M., Kajiyama M., Takemura A., Ono H., Mizumachi H., Hayashi S. (1997). Miscibility between Natural Rubber and Tackifiers. I. Phase Diagrams of the Blends of Natural Rubber with Rosin and Terpene Resins. J. Appl. Polym. Sci..

[34] Kawahara S., Akiyama S. (1993). UCST Phase Behavior and the Miscibility Valley in Blends of Poly(Vinyl Ethylene-Co-1,4-Butadiene) and Hydrogenated Terpene Resin. Macromolecules.

[35] Do Q.-V., Yamaguchi M., Tada T., Doan V.A. (2025). Segregation Behavior of a Tackifier in Styrene-Butadiene Rubber under a Temperature Gradient. Polym. J..

[36] Vincent A.B.A.D., Compagnie Generale des Etablissements Michelin SCA (2020). Rubber Composition Comprising a Styrene-Butadiene Copolymer Having a Low Glass Transition Temperature, and a High Content of Filler and of Plasticizer. U.S. Patent.

[37] Lyulin A.V., Balabaev N.K., Michels M.A.J. (2003). Molecular-Weight and Cooling-Rate Dependence of Simulated Tg for Amorphous Polystyrene. Macromolecules.

[38] Vollmayr K., Kob W., Binder K. (1996). How Do the Properties of a Glass Depend on the Cooling Rate? A Computer Simulation Study of a Lennard-Jones System. J. Chem. Phys..

[39] Brüning R., Samwer K. (1992). Glass Transition on Long Time Scales. Phys. Rev. B.

[40] Han J., Gee R.H., Boyd R.H. (1994). Glass Transition Temperatures of Polymers from Molecular Dynamics Simulations. Macromolecules.

[41] Capponi S., Alvarez F., Račko D. (2020). Free Volume in a PVME Polymer–Water Solution. Macromolecules.

[42] Takahashi K.Z. (2023). Mapping Positron Annihilation Lifetime Spectroscopy Data of a Polymer to Classical Molecular Dynamics Simulations without Shifting the Glass Transition Temperature. J. Chem. Phys..

[43] Riedel C., Alegría A., Tordjeman P., Colmenero J. (2009). Rouse-Model-Based Description of the Dielectric Relaxation of Nonentangled Linear 1,4-Cis-Polyisoprene. Macromolecules.

[44] Ramírez J., Sukumaran S.K., Vorselaars B., Likhtman A.E. (2010). Efficient on the Fly Calculation of Time Correlation Functions in Computer Simulations. J. Chem. Phys..

[45] Behbahani A.F., Bačová P., Polińska P., Burkhart C., Doxastakis M., Harmandaris V. (2024). Local Viscoelastic Properties and Shear Stress Propagation in Bulk and Confined Polymer Melts and Low-Molecular Weight Liquids. Phys. Rev. Res..

[46] Wolf A., Fernandes J.P., Yan C., Dieden R., Poorters L., Weydert M., Verge P. (2021). An Investigation on the Thermally Induced Compatibilization of SBR and α-Methylstyrene/Styrene Resin. Polymers.

