# Supplementary material for: Investigating the Role of Miscibility in Hydrogenated Dicyclopentadiene Resin/Polymer Blends: A Molecular Dynamics Study
Source: Polymers (Basel). 2026 Feb 28;18(5):594. doi: 10.3390/polym18050594 (PMC12987230; doi:10.3390/polym18050594)
Supplement: Supplementary file 1 [file polymers-18-00594-s001.zip › polymers-4101392-supplementary.pdf]

Supplementary Material

Investigating the Role of Miscibility in Hydrogenated Dicyclopentadiene Resin/Polymer Blends: A Molecular Dynamics Study

Anastassia N. Rissanou [1,*], Rohit Ghanta [2], Manolis Doxastakis [2] and Vagelis Harmandaris [3,4,5]

[1] Institute of Theoretical and Physical Chemistry, National Hellenic Research Foundation, 48 Vassileos Konstantinou Ave, 11635 Athens, Greece
[2] Department of Chemical and Biomolecular Engineering, University of Tennessee, Knoxville, TN 37996, USA;
[3] Computation-Based Science and Technology Research Center, The Cyprus Institute, Nicosia 2121, Cyprus;
[4] Department of Mathematics and Applied Mathematics, University of Crete, 70013, Heraklion, Greece
[5] Institute of Applied and Computational Mathematics, Foundation for Research and Technology Hellas, IACM/FORTH, 70013 Heraklion, Greece
* Correspondence: author: trissanou@eie.gr

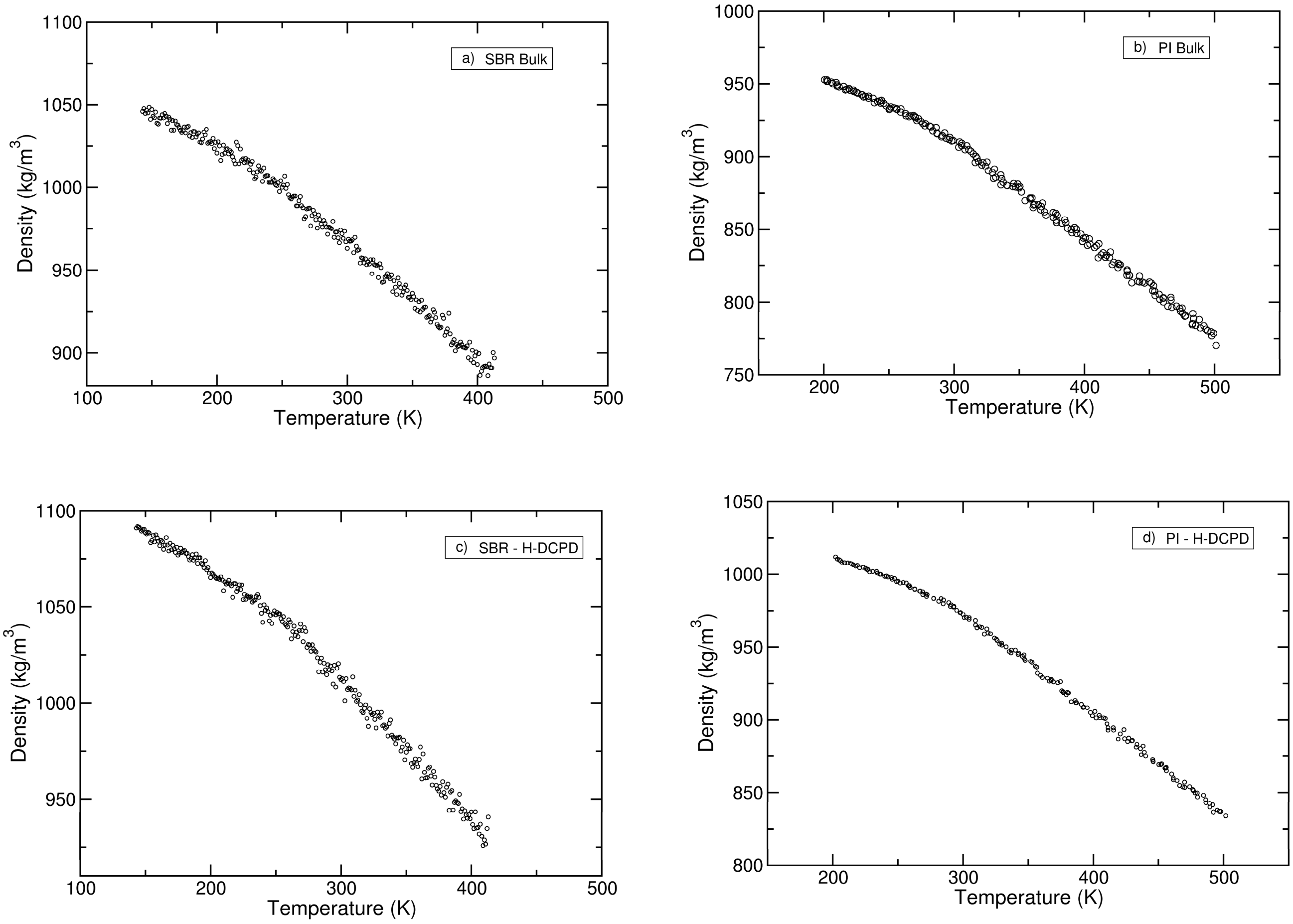

Figure S1: Density as a function of temperature from the quenching runs for a) Bulk SBR, b) Bulk PI, c) SBR – Resin and d) PI – Resin systems.

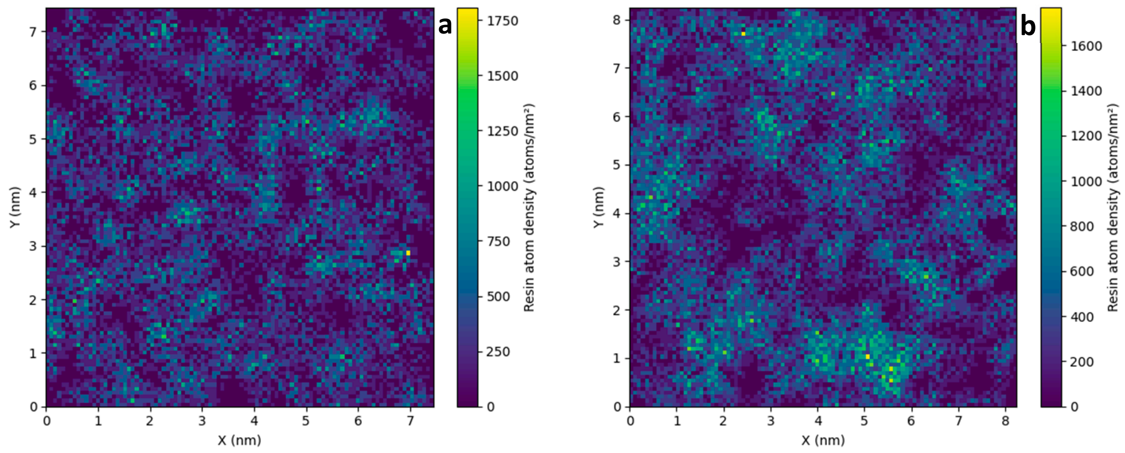

Figure S2: Number density of the resin atoms (atoms/nm^3) (a) at 300K for the SBR - H-DCPD mixture and (b) at 298K for the PI - H-DCPD mixture.

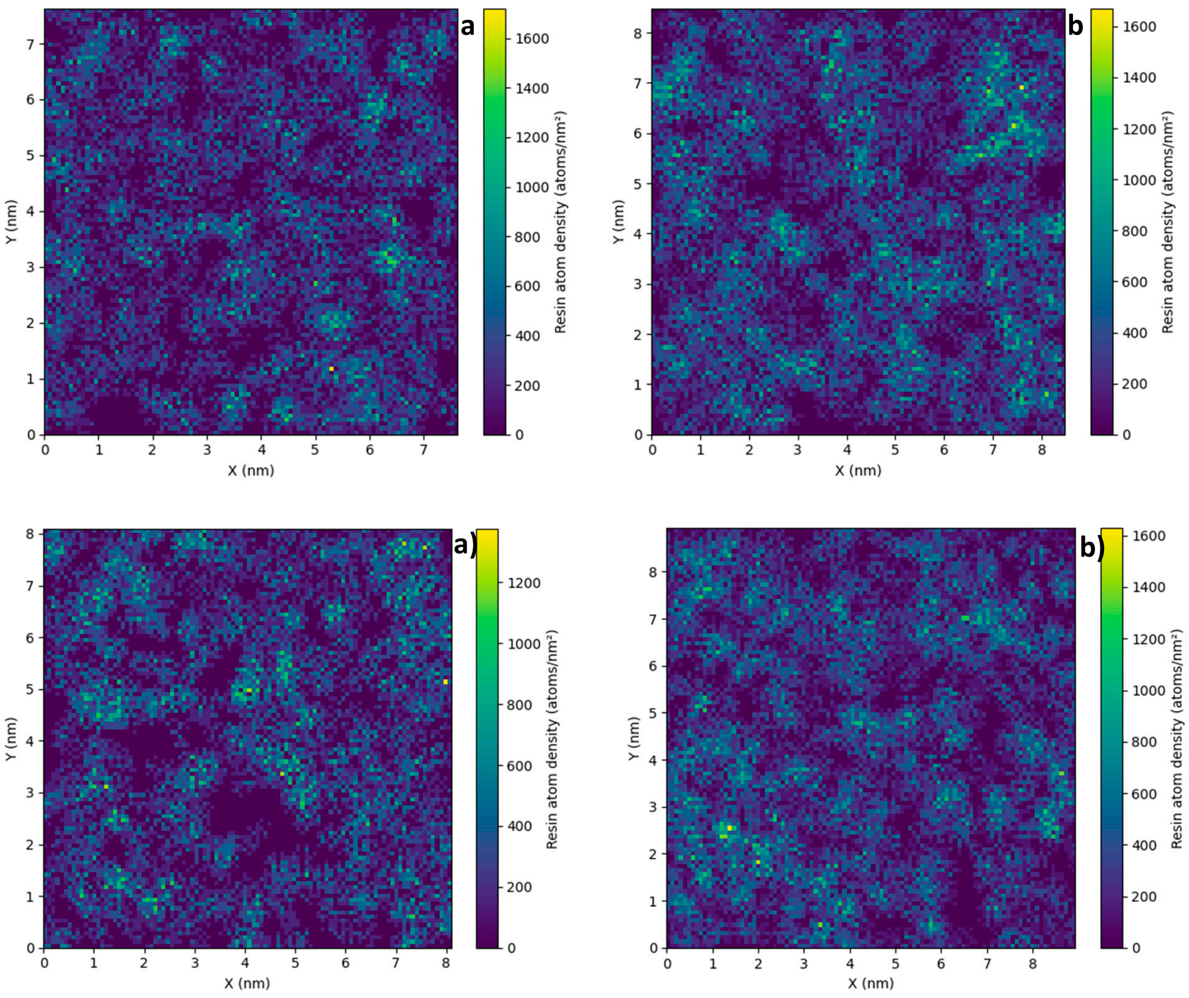

Figure S3: Number density of the resin atoms (atoms/nm^3) for (a) the SBR - H-DCPD mixture and (b) for the PI - H-DCPD mixture at 413K the upper panel and at 600K the lower panel.

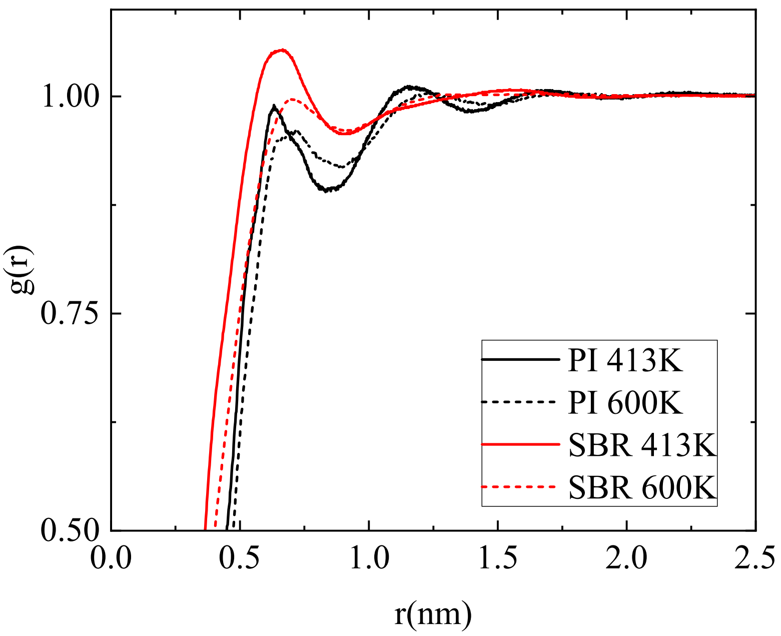

Figure S4: Intermolecular pair radial distribution functions between polymer and resin atoms for PI - H-DCPD and SBR - H-DCPD blends at 413K and 600K.
